# Cognitive Bias as a Mediator in the Relation Between Fear-Enhancing Parental Behaviors and Anxiety Symptoms in Children: A Cross-Sectional Study

**DOI:** 10.1007/s10578-016-0655-2

**Published:** 2016-06-10

**Authors:** Lorraine Fliek, Pauline Dibbets, Jeffrey Roelofs, Peter Muris

**Affiliations:** 1 Clinical Psychological Science, Faculty of Psychology and Neuroscience, Maastricht University, P.O. Box 616, 6200 MD Maastricht, The Netherlands; 2 Stellenbosch University, Stellenbosch, South Africa

**Keywords:** Children’s anxiety symptoms, Cognitive biases, Parenting, Modeling, Threat information transmission

## Abstract

The present cross-sectional study explored the relations between fear-enhancing parenting behaviors (modeling and threat information transmission) and children’s cognitive biases and anxiety symptoms. Participants were 258 children aged 7–12 years (132 boys and 126 girls), and their mothers (*n* = 199) and/or fathers (*n* = 117). Children and parents completed the Parental Enhancement of Anxious Cognitions questionnaire, which measures parental modeling and threat information transmission, while children also filled in a scale for assessing anxiety symptoms. In addition, children conducted a number of computerized tasks for measuring confirmation and interpretation bias. The data indicated that both biases mediated the relationship between threat information transmission (of both parents) and children’s anxiety symptoms. Only interpretation bias significantly mediated the relationship between modeling (of mothers) and anxiety symptoms. These findings give partial support for the hypothesis that cognitive biases play a mediating role in the relation between fear-enhancing parental behaviors and children’s anxiety symptoms.

## Introduction

Fear and anxiety are normal, mild, and transient phenomena in childhood, but in a minority of children these symptoms become so intense and invalidating that they qualify as an anxiety disorder [1]. Heritability is thought to be involved in the etiology of childhood fear and anxiety problems, with behavioral-genetic studies showing that about 30 % of the variation in anxiety disorders can be ascribed to genetic influences [2]. This means that environmental factors also play an important role in the development of fear and anxiety problems, and among these factors parenting behaviors are considered as particularly relevant [3].

In the current study, both parents were included, while previous studies have mostly included only mothers. The role of fathers has often been neglected, although there is some evidence suggesting that fathers play a unique and often different role than mothers in the development of anxiety problems in children (e.g., [4, 5]). In the article we further focus on two types of parenting behaviors that, according to Rachman [6, 7], are involved in the acquisition of fear and anxiety symptoms within the context of the family. The first type is known as modeling or vicarious learning and refers to the phenomenon of children learning anxious behavior after watching parents acting in a fearful way when facing certain stimuli and situations. The second type is threat information transmission, which is concerned with parents installing fear and anxiety in their offspring by telling their children about the dangerousness of particular stimuli and situations. There is a steadily growing body of evidence showing that parental modeling and threat information transmission can promote fear and anxiety in children (see reviews by [8, 9]).

Early studies have typically relied on self-report questionnaires and interviews asking children about these types of learning experiences in relation to their main fear. For instance, in the studies conducted by Ollendick and King [10] and Muris et al. [11], children were first asked to identify their main object of fear, after which they had to indicate to what extent modeling and threat information transmission had played a role in the origins of that fear. It was found that substantial percentages (i.e., 50–90 %) of the children reported such learning experiences, and although their context was not explicitly examined, it is highly plausible that parents were involved.

Subsequent investigations have explored the role of modeling and threat information transmission using experimental designs. With regard to modeling, an exemplary study was conducted by Gerull and Rapee [12] who investigated the influence of fearful responses of mothers to an unknown stimulus on young children’s behavior. Fifteen- to 20-months-old toddlers were confronted with rubber toy animals (i.e., spider, snake), while their mother maintained either a negative or a positive facial expression. After a brief delay, children were again exposed to the toy animals to measure fear and avoidance reactions, this time without their mother being present. The results clearly indicated that toddlers for whom the toy animals had been previously paired with a negative facial expression of their mother showed more fear and avoidance than the toddlers for whom the toy animals had been presented with a positive facial expression of their mother (see also [13]).

To directly examine the effects of threat information transmission, Muris et al. [14] adopted a comparable approach. Parents of children aged 8–13 years (*N* = 88) were presented with either negative, positive, or ambiguous information about an unknown animal and were then given a number of open-ended vignettes describing hypothetical confrontations with the animal. Parents were instructed to tell their children what would happen in these situations. Results indicated that children’s fear levels were influenced by the type of information that was provided to the parent. That is, parents who had received negative information provided more threatening narratives about the animal and hence installed higher levels of fear in their child than parents who had received positive information. In the case of ambiguous information, the transmission of fear was dependent on parents’ trait anxiety levels. More precisely, the higher the trait anxiety level of the parents, the more they were inclined to tell negative stories about the unknown animal on the basis of the ambiguous information, thereby producing higher fear levels in the child. Several other studies have also shown that cognitive biases can be induced in non-anxious individuals by providing them with negative information [15–18].

Thus, there appears to be considerable evidence from both survey and experimental research for the idea that young children can rapidly acquire fear and anxiety via the parental behaviors of modeling and threat information transmission. However, little is known about the mechanisms involved in these ways of fear acquisition. It is well-known that threat-related cognitive biases are a robust correlate of anxiety pathology in children and adults (see reviews by [19, 20]). However, only recently studies have begun to explore the possibility that cognitive biases are involved in the intergenerational transfer of fear and anxiety. An investigation by Lester et al. [21] found evidence indicating that the anxiety-related interpretation bias of mothers was not only concerned with self-referent situations, but also with situations that involved their children, suggesting that mothers may extend their own catastrophic cognitive style to the living environment of the children. Another study by Podină et al. [22] took this one step further and actually investigated whether cognitive biases indeed acted as mediators between maternal social anxiety and children’s anxiety symptoms. Four-hundred-and-twenty-three mothers and their children completed questionnaires measuring anxiety symptoms and interpretation bias. Multiple mediation analysis demonstrated that both maternal and child interpretation biases were significant mediators in the relation between maternal social anxiety and children’s anxiety symptoms. In similar research by Affrunti and Ginsburg [23], it was also demonstrated that interpretation biases acted as the connector between parental and children’s anxiety symptoms. A final relevant investigation was recently conducted by Remmerswaal et al. [24] who examined the role of mothers in the development of a cognitive bias and subsequent fear levels in their offspring. Using an inventive experimental design, these researchers were able to show that mothers induced a negative information search bias in their children either on the basis of instruction or driven by their own anxiety, which was also associated with heightened fear levels in relation to a novel stimulus.

The above described research provides support for the idea that cognitive biases play a role in the transfer of fear and anxiety from parents to offspring. The aim of the present cross-sectional study was to further contribute to this literature. In a sample of 258 non-clinical youths aged 9–12 years, fear-enhancing parental variables (i.e., modeling and threat information transmission), children’s cognitive biases, and children’s and parents’ anxiety symptoms were measured. In line with Hadwin et al. [25] who claim that cognitive biases might have their origins in parenting, we hypothesized that these biases would act as a mediator in the link between the parenting behaviors of modeling and threat information transmission and children’s anxiety symptoms.

The current study adds to our existing knowledge in four ways: (1) A newly developed questionnaire was used which made it possible to simultaneously examine both modeling and threat information transmission as predictors of children’s cognitive biases and subsequent anxiety symptoms. (2) The fear-enhancing parental behaviors of both parents were investigated, which can be seen as an advancement to previous studies that mainly focused on the role of mothers (e.g., [4]). Note that the comparison between both parents was exploratory in nature and so we did not have an explicit hypothesis. (3) Two types of cognitive biases, interpretation bias and confirmation bias, were assessed. Interpretation bias refers to the inclination to infer threat on the basis of ambiguous information, whereas confirmation bias has to do with the tendency to search for information that confirms one’s anxious preconceptions, while ignoring information that could disconfirm threat. Most research has focused on only one type of bias, thereby neglecting the issue of whether such biases are inter-related and make independent contributions to anxiety. Although both interpretation bias and confirmation bias seem to be linked to the interpretation stage of social information processing [26], the present study explored the unique role of both biases as mediators in the relation between fear-enhancing parenting behaviors and children’s anxiety symptoms. (4) Because both parents also completed measures of trait anxiety and overprotection, we were also able to investigate to what extent the fear-enhancing parental behaviors of modeling and threat information transmission were associated with these two well-established parental correlates of childhood anxiety problems [27, 28]. Based on previous studies we expected that higher levels of fear-enhancing parenting behaviors would be associated with higher levels of trait anxiety and overprotection of parents.

## Method

### Participants

Participants were 258 non-clinical children (132 boys and 126 girls) aged between 7 and 12 years (*M* = 9.52, *SD* = 1.38) and their parents. A total of 199 mothers and 117 fathers (mean ages being 42.20 years, *SD* = 4.42 and 44.36 years, *SD* = 4.95, respectively, range 28–65 years) also participated in this study. All children had the Dutch nationality and the majority of them were from original Dutch descent (>95 %). The remainder of the families represented a diversity of nationalities (i.e., German, Belgian, American, Moroccan, Irish, Hungarian, Swedish, and Iraqi). Parental questionnaires were nearly always completed by children’s biological parents; the two exceptions were one child who had adoptive parents and one child who was raised by two mothers. The latter child only answered the questions with regard to his biological mother. About 15 % of children came from divorced families.

### Child Measures

The *Parental Enhancement of Anxious Cognitions* (PEAC) was construed for the purpose of this study. Initially, 23 items were created that referred to the fear-enhancing parental behaviors of modeling and verbal threat information. Two steps were taken to obtain a final version of the scale that we considered as appropriate for our research. The first step involved an inspection of the initial set of PEAC items by two research experts in the field of fear acquisition (prof. Andy Field of Sussex University, Brighton, United Kingdom, and prof. Stanley Rachman of the University of British Columbia, Vancouver, Canada) and 26 clinicians who worked with anxiety disordered children. The two experts helped us to refine and improve the items of the questionnaire, while the clinicians performed a face validity check: they were asked to classify each of the 23 PEAC items as either modeling or threat information transmission. The face validity check was satisfactory: clinicians classified almost all items correctly to either modeling or threat information transmission. The 2 threat information items that were not correctly classified by more than 2 clinicians were removed; these were 2 negatively formulated items and it appeared that they did not load consistently on the two factors. The second step was an exploratory factor analysis (with direct oblimin rotation), which was performed on the PEAC data of the children and their parents.^1^ For children, fathers, and mothers, the factor analysis produced the hypothesized structure with one factor representing modeling behaviors and one threat information transmission. However, 9 items (predominantly negatively formulated items) had to be removed as they did not load consistently on one of the two factors across the three informants. Thus, eventually 14 items were retained in the final version of the PEAC: 6 items pertained to modeling, while 8 items were concerned with threat information transmission (see Appendix). The child version of the PEAC asks children for each item to first rate the frequency of their fathers’ and then that of their mothers’ fear-enhancing behaviors, using 4-point Likert-scales (0 = never, 1 = sometimes, 2 = often, and 3 = always). For each factor, a total score can be computed by summing the ratings on relevant items. In the current study, Cronbach’s alphas of the child version of the PEAC modeling and threat information transmission factors were .65 and .80 for the mother scales and .66 and .84 for the father scales, indicating that the measure has sufficient to good reliability.

The Revised version of the *Screen for Child Anxiety Related Emotional Disorders* is an extension of the original SCARED [29, 30] and assesses symptoms of the entire spectrum of DSM-IV-defined [31] anxiety disorders in children and adolescents. In the current study only the SCARED subscales of social phobia (7 items; e.g., “I don’t like to be with unfamiliar people”), generalized anxiety disorder (9 items; e.g., “I worry about things working out for me”), and separation anxiety disorder (8 items; e.g., “I don’t like being away from my family”) were used, because these three types of anxiety were considered as most relevant for the scenarios that were employed to assess cognitive biases. Children were asked to rate the frequency with which they experienced each symptom using a three-point scale (0 = almost never, 1 = sometimes, 2 = often) and a total anxiety score can be obtained by summing ratings on the items of the three selected anxiety scales (range 0–48). Research has demonstrated that the SCARED-R has good internal consistency, test-retest reliability, and validity [30, 32, 33]. In the current study, the mean total anxiety score on the shortened SCARED was 16.67 (*SD* = 8.21) and an independent samples *t*-test revealed that girls (*M* = 18.29, *SD* = 7.94) scored higher on this scale than did boys (*M* = 15.14, *SD* = 8.20) [*t*(256) = 3.13, *p* < .01]. Further, the reliability of the SCARED-R total anxiety score was good, with a Cronbach’s alpha of .87.

*The Information Search Task* (IST) was also developed for the purpose of this study to assess children’s confirmation bias. The task was based on a similar paradigm as used in a previous study [34]. Children were presented with new, potentially threatening situations (e.g., going to a new school) about which they had to gain more information (e.g., “What would you like to know about the teachers at your new school?”) by choosing between a positive (e.g., “Whether they have a nice way of teaching”) and a negative (e.g., “Whether they become angry very easily”) option. After making their choice, children always received a confirmative answer (e.g., positive: “Most teachers have a nice way of teaching”, negative: “Most teachers become angry very easily”). In total, children were presented with 3 situations (the other two scenarios were: going to the new warehouse in the city and playing at a friend’s home for the first time), for each of which they were able to seek new information 5 times. The types of information the children could collect were related to the different types of anxiety symptoms. A confirmation bias score was computed by summing the number of negative options chosen (range 0–15). The reliability of the IST was acceptable, with a Cronbach’s alpha of .72.

To assess interpretation bias, we used three *Ambiguous Stories* [35], which represented the themes of social anxiety (i.e., going to a sporting club for the first time), generalized anxiety (i.e., driving with your bike on a very busy street), and separation anxiety (i.e., staying with a friend while parents are on vacation). Children had to read the stories, which consisted of five sentences presented to them sentence by sentence on the computer screen. Following each sentence, they were asked whether they thought that the story would be “scary” or “not scary”. A total interpretation bias score was calculated by summing up the number of sentences after which children indicated the story was going to be scary (range 0–15). The reliability of the Ambiguous Stories test was sufficient, with a Cronbach’s alpha of .63.

### Parent Measures

The Y2-version of the *Spielberger State-Trait Anxiety Inventory* (STAI; [36, 37]) was used to assess trait anxiety in the parents. The questionnaire includes 20 items (e.g., “I feel nervous” and “I worry too much about little things”) for which respondents have to indicate their answer on a four-point Likert type scale (1 = almost never, 2 = sometimes, 3 = often, 4 = almost always). After recoding the positively phrased items, a total score can be obtained by summing all items (range 20–80). There is clear support for the psychometric properties of the STAI [36, 38]. Cronbach’s alphas in the present sample were .92 for the mothers and .94 for the fathers, indicating excellent reliability.

The *Parental Overprotection Measure* (POM; [39]) was used to measure overprotective parenting behaviors which are thought to restrict the child’s exposure to situations that are perceived as threatening or harmful. The instrument consists of 19 items (e.g., “I do not allow my child to climb in trees” and “I protect my child from criticism”) that are scored on a five-point Likert scale (0 = not at all, 1 = a little, 2 = somewhat, 3 = quite often, 4 = very often). A total score can be obtained by summing all items (range 0–76). The scale has been shown to possess high internal consistency, strong test-retest reliability, and good validity [39]. In the current study reliability was also good, with Cronbach’s alphas of .88 for the mothers and .87 for the fathers.

A parent version of the *Parental Enhancement of Anxious Cognitions (PEAC)* was also completed by the parents. The scale is similar to the one completed by the children, but the 14 items are formulated from the perspective of the parent (e.g., “I warn my child explicitly that he/she should avoid dangerous situations” instead of “My mother/father warns me explicitly that I should avoid dangerous situations”). Cronbach’s alphas of the modeling and threat information transmission factors were .77 and .81 for the mother scales and .68 and .72 for the father scales, indicating that the parent version of the PEAC has sufficient to good reliability.

### Procedure

Participants were recruited via four Dutch primary schools. Informed parental consent was obtained by sending parents an information letter about the study with a consent form. Children for whom parents granted permission were tested in small groups (of approximately eight children per group) in a separate room in school. Each child used a computer to fill out the questionnaires and to conduct the cognitive bias tasks. This assessment took place under supervision of two experimenters, who guided the children through the session by providing instructions and by collectively conducting some practice items. The children received explicit instructions to call upon the experimenters in case they had any questions about the scales or tasks. The experimenters ensured that the children answered the questions and conducted the computer tasks confidentially and independently. Children first completed the SCARED and the PEAC, after which they carried out the IST and the Ambiguous Stories test. Children did not appear to experience great difficulties while completing the questionnaires and computer tasks. Some children had questions about how to fill out the negatively formulated items of the PEAC (most of these items were eventually removed during the factor analyses). Parents completed the questionnaires at home on their own computer using a web-link provided to them by the experimenters.

### Statistical Analyses

*T*-tests and correlations were computed to investigate possible differences and links among various child and parent scales. To investigate whether the two cognitive biases acted as mediators in the relation between modeling and threat information transmission on the one hand and anxiety symptoms of the child on the other hand, we conducted a bootstrapping procedure for multiple mediators [40] using the child-report data. We used the SPSS macro relying on a method with 1000 bootstrap resamples to test the indirect effects of modeling and threat information transmission via the potential mediating variables on child anxiety. The output provides a 95 % confidence interval of the indirect effects, controlling for the effects of the other variables. If zero is not included in the confidence interval, the effect is considered significant. This means that the effect of the independent variable (modeling or threat information) on the dependent variable (child anxiety) is mediated by the proposed mediators (confirmation bias or interpretation bias). We conducted four different analyses: one for each independent parenting variable of mothers and fathers.

## Results

### General Results

Before discussing the main findings of the present study, a number of general results will be addressed. First, *t*-tests comparing the levels of modeling and threat information transmission between fathers and mothers revealed significant differences when these parenting behaviors were assessed from the child’s perspective. As can be seen in Table 1, children indicated that their mothers more often displayed modeling [*t*(248) = 9.39, *p* = .01] and threat information transmission [*t*(248) = 9.29, *p* = .01] than their fathers. When using the parents’ point-of-view, no significant differences between fathers and mothers were observed, although a trend was noted signaling somewhat higher levels of threat information transmission in fathers than in mothers [*t*(105) = 1.93, *p* = .06]. No differences were found between fathers and mothers with regard to trait anxiety or overprotective parenting [both *t*(106)’s < 1; see Table 1].

**Table 1:** Mean scores (standard deviations) on parent-related questionnaires as completed by parents and children separately, as well as reliability coefficients for various scales

	Mothers	α	Fathers	α
Children				
PEAC modeling	10.77 (3.14)^a^	.65	9.39 (2.82)^b^	.66
PEAC threat info	22.94 (4.96)^a^	.80	21.14 (5.44)^b^	.84
Parents				
PEAC modeling	9.40 (2.69)^b^	.77	9.19 (2.46)^b^	.68
PEAC threat info	20.24 (4.27)^b^	.81	20.97 (3.54)^b^	.72
STAI trait anxiety	31.90 (8.12)	.92	31.86 (8.88)	.94
POM overprotection	24.12 (10.40)	.88	25.06 (10.08)	.87

*N*’s were 199 for mothers, 117 for fathers and 258 for children

^a, b^For each type of fear-enhancing parenting behavior, within-row and within column means not sharing similar superscripts differ at *p* < .01

*STAI* State-Trait Anxiety Inventory, *POM* Parental Overprotection Measure, *PEAC* Parental Enhancement of Anxious Cognitions, *Threat info* Threat information transmission

Second, a statistical comparison of the PEAC scores of children and parents indicated that children scored their mothers as higher on both modeling [*t*(198) = 5.70, *p* < .001] and threat information transmission [*t*(198) = 6.43, *p* < .001] than mothers themselves (Table 1). No such differences were observed when comparing the PEAC scores between children and fathers [both *t*(115)’s ≤ 1.56, *p’*s ≥ .12].

### Correlation Analysis

Table 2 displays correlations among various child and parent measures included in this study. A number of conclusions can be drawn from this table. When looking at the correlations obtained among the child measures, it can first of all be concluded that fear-enhancing parenting behaviors of fathers and mothers were positively and significantly correlated (all *r*’s between .31 and .83, *p*’s < .01). Thus, according to the children, mothers’ and fathers’ modeling and negative information transmission were moderately to strongly associated. Fear-enhancing parenting behaviors were also positively related to children’s anxiety symptoms (*r*’s between .32 and .37, *p*’s < .01), implying that the more children perceived modeling and negative information transmission in fathers and mothers, the higher their levels of anxiety symptoms. Further, children’s anxiety symptoms were also positively linked to cognitive bias scores (*r*’s = .31 and .49, *p*’s < .01). That is, higher levels of anxiety symptoms were accompanied by higher levels of threatening interpretations of ambiguous stories (i.e., interpretation bias) and a stronger tendency to search for negative information (i.e., confirmation bias). Finally, a small but significant positive correlation was found between both types of cognitive biases (*r* = .20, *p* < .01), meaning that a stronger inclination towards threat interpretation was also to some extent associated with a stronger tendency to search for negative information.

**Table 2:** Correlations among all child and parent measures

	(1)	(2)	(3)	(4)	(5)	(6)	(7)	(8)	(9)	(10)	(11)	(12)	(13)	(14)
Child														
1. Confirmation bias														
2. Interpretation bias	.20**													
3. PEAC modeling M	.18**	.27**												
4. PEAC modeling F	.17**	.20**	.70**											
5. PEAC threat info M	.33**	.25**	.47**	.37**										
6. PEAC threat info F	.29**	.26**	.31**	.44**	.83**									
7. SCARED total anxiety	.31**	.49**	.37**	.36**	.33**	.32**								
Mother														
8. STAI Trait anxiety	.06	.02	.00	−.03	.00	−.04	.02							
9. POM overprotection	.00	.05	.14*	.16*	.08	.06	.12	.20**						
10. PEAC Modeling	−.05	.06	.25**	.06	.09	−.08	.11	.28**	.20**					
11. PEAC threat info	.07	.04	.14*	.11	.15*	.10	.17*	.14*	.60**	.40**				
Father														
12. STAI trait anxiety	−.13	−.03	.00	.11	−.16	−.12	−.06	.20*	−.02	−.05	−.12			
13. POM overprotection	.04	−.14	−.11	.03	.09	.15	.05	.10	.38*	−.13	.10	.06		
14. PEAC modeling	.04	−.03	.16	.28**	.09	.10	.03	−.08	−.04	.12	.04	.23*	.06	
15. PEAC threat info	.09	−.04	−.12	−.08	.11	.13	−.05	−.01	.17	−.08	.15	.04	.55**	.14

*N*’s were 199 for mothers, 117 for fathers and 258 for children

*PEAC* Parental Enhancement of Anxious Cognitions, *Threat info* threat information transmission, *M* child about mother, *F* child about father, *SCARED* Screen for Child Anxiety Related Disorders, *STAI* State-Trait Anxiety Inventory, *POM* Parental Overprotection Measure

* *p* < .05, ** *p* < .01

The correlations among the scales as completed by the mothers revealed the expected pattern of positive links among trait anxiety, overprotection, and the fear-enhancing parental behaviors of modeling and negative information transmission. Most of these correlations were in the small to moderate range, but in particular the correlation between overprotection and threat information transmission was quite robust (*r* = .60, *p* < .001). In fathers, the correlations among parental indices were less clear. Only a small correlation between trait anxiety and modeling was found (*r* = .23, *p* < .05), whereas the link between overprotection and threat information transmission was again substantial (*r* = .55, *p* < .001).

When looking at the cross-informant correlations in Table 2, the overall conclusion is that few significant associations between child- and parent-reports and between measures of mothers and fathers were found. As for fear-enhancing parenting, significant correlations emerged for modeling of mothers and fathers as reported by the children and modeling as reported by mothers and fathers themselves (*r*’s being .25 and .28, respectively, *p*’s < .01). In addition, threat information transmission of mothers as reported by the children was positively linked to threat information transmission as reported by mothers themselves, although the magnitude of this correlation was small (*r* = .15, *p* < .05). Fathers and mothers did show some similarity with regard to their level of trait anxiety and overprotective parenting (*r*’s being .20 and .38, *p*’s < .05), but for the fear-enhancing behaviors of modeling and threat information transmission correlations between fathers and mothers were non-significant. Finally, parental indices and children’s anxiety symptoms and cognitive biases were largely unrelated, except for a small positive correlation between threat information transmission of mothers and children’s anxiety symptoms (*r* = .17, *p* < .05).

### The Mediating Role of Cognitive Biases

The results of the mediation analyses are presented in Table 3. In the first analysis, threat information transmission of mothers was the independent variable. The indirect effects of confirmation bias (bias corrected 95 % CI = .02, .10) and interpretation bias (bias corrected 95 % CI = .01, .12) were both significant. The direct effect was not significant, which implies that these cognitive biases mediated the relationship between maternal threat information transmission and child anxiety. In the second analysis, modeling of mothers was the independent variable. Only the indirect effect of interpretation bias was found to be significant (bias corrected 95 % CI = .03, .14). Here, the direct effect still accounted for a significant proportion of the variance in children’s anxiety symptoms.

**Table 3:** Results of the bootstrapping analyses testing cognitive biases as mediators between child-reported threat information transmission and modeling of parents and children’s anxiety symptoms

IV	M	Effect of IV on M	Effect of M on DV	Direct effect	Indirect effect	Total effect
Mothers						
Threat information	Confirmation bias	.31***	.17**	.09	.05ª	.20**
	Interpretation bias	.16*	.39***		.16ª	
Modeling	Confirmation bias	.03	.17**	.19**	.01	.28***
	Interpretation bias	.20**	.39***		.08ª	
Fathers						
Threat information	Confirmation bias	.26***	.18**	.06	.05ª	.20**
	Interpretation bias	.21**	.42***		.09ª	
Modeling	Confirmation bias	.05	.18**	.21***	.01	.27***
	Interpretation bias	.11	.42***		.05	

*IV* independent Variable, *M* mediator, *DV* dependent variable

^a^Significant point estimate (*p* < .05)

* *p* < .05, ** *p* < .01, *** *p* < .001

In the third analysis, threat information transmission of fathers was the independent variable. Both the indirect effects of confirmation bias (bias corrected 95 % CI = .02, .09) and interpretation bias (bias corrected 95 % CI = .03, .16) were significant. As the direct effect was non-significant, it can be concluded that both cognitive biases mediated the relationship between paternal threat information transmission and child anxiety. In the final analysis, modeling of fathers was the independent variable. The indirect effects for confirmation bias (bias corrected 95 % CI = −.01, .04) and *interpretation bias* (bias corrected 95 % CI = −.01, .11) were not significant, which means that there was no indication for a mediation effect.

## Discussion

The aim of the present study was to examine whether cognitive biases play a role in the transfer of fear and anxiety from parents to offspring. More specifically, it was investigated whether confirmation bias and interpretation bias would act as mediators in the relation between the fear-enhancing parenting behaviors of modeling and threat information transmission and anxiety symptoms in children. The results first of all showed that child-reported modeling and threat information transmission of fathers and mothers were positively related to children’s anxiety symptoms. Thus, children who indicated that their parents often acted as an anxious model or frequently communicated threat information also displayed higher levels of anxiety. Further, evidence was found indicating that both confirmation and interpretation bias mediated the relationship between threat information transmission of both mother and father and children’s anxiety symptoms, which is in keeping with other studies investigating the mediational role of cognitive bias in the relation between parenting behaviors and childhood anxiety problems [41–43]. The support for a mediating role of cognitive bias in the relation between modeling and children’s anxiety symptoms was less convincing. Only interpretation bias mediated the relationship between modeling of mothers (but not of fathers) and children’s anxiety symptoms. Remarkably, the direct effect of modeling on children’s anxiety symptoms still accounted for a significant proportion of the variance in the models of both the mothers and the fathers. This implies that parental modeling does not solely exert its impact via children’s cognitive biases, but suggests other mediators (or moderators) to be involved in the relation between maternal modeling and children’s anxiety symptoms.

It is unclear why cognitive biases played a more important role in the relation between parental threat information transmission and children’s anxiety symptoms than it did in the relation between parental modeling and children’s anxiety symptoms. A plausible explanation has to do with the fact that cognitive biases are verbal in nature, and this might be the *modus operandi* of threat information transmission. That is, parents verbally communicate threat information to the child, thereby having a direct impact on the cognitions of their offspring. The mechanism involved in the relation between modeling and child anxiety seems to be quite different. Illustrative in this regard is the research on social referencing; children observe their mothers reacting with fear and anxiety to a certain stimulus or situation, and subsequently start to copy that anxious behavior when they are exposed to that stimulus or situation themselves [44]. This mechanism already occurs in children at a young age and seems less cognitive, but above all emotional-behavioral in nature.

The pattern of the effects in the mediation models was more or less comparable for both parents. It may well be the case that the contributions of fathers and mothers to children’s anxiety symptoms are made via the same mechanisms. However, one should also be aware of the possibility that similarities in findings for mothers and fathers might have been mainly due to the fact that these analyses only relied on the child-report data, for which the mother and father ratings on the PEAC were highly correlated. The cross-informant (i.e., child-father, child-mother) correlations in general yielded few significant findings (see review by [45]), and thus were less suitable for studying differential findings between both parents. It would have been interesting if we had assessed all constructs (that is, not only fear-enhancing parenting behaviors, but also children’s cognitive biases and anxiety symptoms) from both parents’ point-of-view, and this is an important venue for further research.

When looking at the PEAC data, a number of additional findings should be noted. First, according to the children, mothers displayed more modeling and threat information transmission than fathers, which is of course in keeping with the literature indicating that females (mothers) generally display higher levels of fear and anxiety and are probably more likely to engage in fear-enhancing behaviors than males [46]. However, when looking at the parental data, no evidence was found for this idea: that is, mothers and fathers did not only report equal levels of modeling and threat information transmission, but also did not differ in terms of trait anxiety and overprotection. It is not clear why children rated their mothers as higher on fear-enhancing behaviors, but one explanation could be that mothers more often act as the primary caretaker and thus spent more time with their children [47]. It is possible that for this reason children could think of more examples when evaluating the behaviors of their mothers and as such provided higher ratings of modeling and threat information transmission. Second, the child-parent and mother-father correlations for the PEAC scales were rather low or non-significant. Again, this is in line with previous findings and indicates that informant discrepancies not only occur when assessing childhood symptoms but also when measuring contextual, etiological factors, such as fear-enhancing parental behaviors. Third, when looking at the relations between the PEAC scales and two well-known parental correlates of childhood anxiety symptoms, namely parental trait anxiety and overprotective parenting [27, 28], the mother data clearly showed the predicted pattern of findings. That is, positive and significant correlations emerged among modeling, threat information transmission, trait anxiety, and overprotection. The father data only revealed significant correlations between trait anxiety and modeling, and between overprotection and threat information transmission. Obviously, these results provide some support for the validity of the PEAC scales. The fact that the most robust correlation (for both mothers and fathers) was found between overprotection and threat information transmission makes sense, and seems to point out that parents especially try to shield their offspring against potential danger by providing them with threatening information.

The two types of cognitive biases, interpretation bias and confirmation bias, were only moderately correlated. The modest overlap between both biases was also confirmed in the mediation analyses, which showed that interpretation bias and confirmation bias each made their own unique contributions as mediators in the relation between parental fear-enhancing behaviors and children’s anxiety symptoms. Since interpretation and confirmation bias are both thought to occur during the more conceptual stages of information processing [20, 26], we had expected to find a higher correlation between these biases. So far, few other studies have explored the relations among (children’s) cognitive biases and the unique links of these biases to anxiety disorders symptoms. One exception is an investigation by Dalgleish et al. [48] who also documented small, mostly non-significant correlations among various types of biases in a sample of youth with mixed internalizing disorders. These authors argued that this may have been primarily due to the fact that these biases are measured with different experimental tasks, thereby introducing quite a large amount of non-shared variance which leads to fairly low inter-correlations among various biases. Obviously, this argument is also true for the two biases that were investigated in the present study.

The assessment of multiple cognitive biases was certainly a strong point of this study as was the inclusion of both mothers and fathers. However, the present investigation also suffers from a number of limitations. To begin with, this was a cross-sectional study, which means that no conclusions about the directionality of the relationships can be drawn. Obviously, a longitudinal set-up is needed, a requirement which will be met as we are planning a follow-up assessment of this sample. A further shortcoming has already been mentioned and is concerned with the fact that we did not take all the assessments in every informant. Moreover, it would have been preferable if we had not only relied on rating scales for measuring modeling and threat information transmission, but had also employed some kind of interview or observational method to assess these fear-enhancing parental behaviors. More specifically, children could either be asked open-ended questions to learn more about how their parents discourage various behaviors or how threatening their parents view the world (e.g., new places, unfamiliar people, risky situations), or child-parent interactions could be observed in challenging situations. Apart from the fact that such a multi-method approach is preferable, this would also give us the opportunity to study the validity of the PEAC more thoroughly. Furthermore, the reliability coefficients of the modeling subscale of the PEAC, in particular for the child and father data, were on the low side. The most plausible explanation might be that the modeling scale consists of only a limited set of items that are quite heterogeneous in terms of content. That is, modeling items refer to parents’ concealed fear reactions, facial expression, body language, and panic symptoms, which may not all be equally well observed and scored, especially by children. Another shortcoming of the study has to do with the fixed sequence in which the questionnaires were administered, which may have introduced some unintended order effects. For example, responding to SCARED social anxiety items may have primed children’s responses on the bias tasks, which were partly geared to assess children’s responses to scenarios depicting social situations. Finally, children’s PEAC scores for mothers and fathers were strongly correlated, which may be the result of the method of scoring each item simultaneously for both parents (left on the screen: father, right on the screen: mother). For future studies, it would be better to present the father and mother versions of this questionnaire serially instead of parallel.

This cross-sectional study is the first to explore relations between parental modeling and verbal threat information and child anxiety symptoms, while taking into account the role of two types of cognitive biases: confirmation bias and interpretation bias. Some evidence was provided for a mediating role of cognitive biases in the relation between threat information transmission and child anxiety. This suggests that intervention programs targeting anxiety problems of children via the parents should target the threat information transmission pathway if one intends to produce cognitive change, whereas a focus on the modeling pathway is required to produce behavioral change. Future research should explore this possibility in anxious children of various ages.

## Summary

Parents are thought to be involved in the etiology of anxiety pathology in children. This cross-sectional study explored whether parental modeling and threat information transmission would be positively related to anxiety symptoms in children. It was hypothesized that this relationship would be mediated by children’s cognitive biases. Participants consisted of 258 children aged between 7 and 12 years and their parents. Children and parents completed the Parental Enhancement of Anxious Cognitions questionnaire, which measures parental modeling and threat information transmission, while children also filled in a scale for assessing anxiety disorder symptoms. In addition, children conducted a number of computerized tasks for measuring confirmation and interpretation bias. Evidence was found for a mediating role of cognitive biases in the relation between threat information transmission and child anxiety, but the support for a mediating role of cognitive bias in the relation between modeling and child anxiety was less convincing. A possible explanation for this finding might be that cognitive biases as well as threat information transmission are more verbal in nature, while the mechanism involved in the relation between modeling and child anxiety seems to be quite different. Modeling is probably more related to social referencing, which is a topic in need of further scientific inquiry. Our data provide more insight in the relations between fear-enhancing parenting behaviors, cognitive biases, and anxiety symptoms in children.

## Appendix

See Table 4.

**Table 4:** Parental Enhancement of Anxious Cognitions (PEAC)

Father					Mother			
Not at all true (0)	Somewhat true (1)	True (2)	Very true (3)		Not at all true (0)	Somewhat true (1)	True (2)	Very true (3)
				1. This parent warns me about potential dangers, to prevent accidents from happening				
				2. When I am anxious to do something, this parent cannot conceal his/her worries				
				3. This parent explicitly warns me that I should be careful when I leave home				
				4. This parent warns me that I should never talk to strangers because of bad things that might happen				
				5. When this parent is scared, his/her body language reveals his/her fear (e.g., fidgeting hands, sweating, trembling, touching neck or face)				
				6. This parent shows when he/she is in panic in my presence				
				7. When this parent is scared, he/she has a fearful expression on his/her face in my presence				
				8. This parent shows me that he/she is afraid to do certain things				
				9. This parent warns me explicitly that I should avoid dangerous situations				
				10. This parent tells me that the world is not always a safe place				
				11. Even if this parent tries to hide his/her fear, I can still see that he/she is anxious				
				12. When I do something new or go to a new place, this parent warns me about the things that could go wrong				
				13. This parent points out to me that an accident can always happen				
				14. This parent warns me explicitly not to go along with unfamiliar people				

Threat information transmission: items 1, 3, 4, 9, 10, 12, 13, 14; Modeling: items 2, 5, 6, 7, 8, 11

## Footnotes

1 These data can be obtained from the first author.
